# Bio-Inspired Controller on an FPGA Applied to Closed-Loop Diaphragmatic Stimulation

**DOI:** 10.3389/fnins.2016.00275

**Published:** 2016-06-16

**Authors:** Adeline Zbrzeski, Yannick Bornat, Brian Hillen, Ricardo Siu, James Abbas, Ranu Jung, Sylvie Renaud

**Affiliations:** ^1^Bordeaux INP, IMS, UMR 5218Talence, France; ^2^Univ. Bordeaux, IMS, UMR 5218Talence, France; ^3^Department of Biomedical Engineering, Florida International UniversityMiami, FL, USA; ^4^School of Biological and Health Systems Engineering, Arizona State UniversityTempe, AZ, USA

**Keywords:** bio-inspired controller, spiking neural network (SNN), closed-loop paradigm, ventilatory control system, metabolic demands, spinal-cord injury (SCI), field programmable gate array (FPGA), assisted ventilation

## Abstract

Cervical spinal cord injury can disrupt connections between the brain respiratory network and the respiratory muscles which can lead to partial or complete loss of ventilatory control and require ventilatory assistance. Unlike current open-loop technology, a closed-loop diaphragmatic pacing system could overcome the drawbacks of manual titration as well as respond to changing ventilation requirements. We present an original bio-inspired assistive technology for real-time ventilation assistance, implemented in a digital configurable Field Programmable Gate Array (FPGA). The bio-inspired controller, which is a spiking neural network (SNN) inspired by the medullary respiratory network, is as robust as a classic controller while having a flexible, low-power and low-cost hardware design. The system was simulated in MATLAB with FPGA-specific constraints and tested with a computational model of rat breathing; the model reproduced experimentally collected respiratory data in eupneic animals. The open-loop version of the bio-inspired controller was implemented on the FPGA. Electrical test bench characterizations confirmed the system functionality. Open and closed-loop paradigm simulations were simulated to test the FPGA system real-time behavior using the rat computational model. The closed-loop system monitors breathing and changes in respiratory demands to drive diaphragmatic stimulation. The simulated results inform future acute animal experiments and constitute the first step toward the development of a neuromorphic, adaptive, compact, low-power, implantable device. The bio-inspired hardware design optimizes the FPGA resource and time costs while harnessing the computational power of spike-based neuromorphic hardware. Its real-time feature makes it suitable for *in vivo* applications.

## Introduction

Approximately 282,000 Americans (~0.1% of the US population) have traumatic spinal cord injury (SCI), with approximately 17,000 new injuries being reported each year (National Spinal Cord Injury Statistical Center—NSCISC, 2016). The cervical cord is the most common site of injury (54% of all cases). An individual with high-level cervical SCI at or above the fifth cervical level may experience partial or complete loss of ventilatory control (Warren et al., 2014) because the motor fibers of the phrenic nerve, which innervates the diaphragm, originate from the 3rd to 5th cervical level. Respiratory diseases and failure are the leading cause of death after SCI (67.4% of these were cases of pneumonia) (NSCISC, 2016). Ventilatory insufficiency is often managed with positive pressure mechanical ventilation, but such treatment is associated with significant discomfort, diaphragmatic atrophy, atelectasis and barotrauma (DiMarco, 2005). Most mechanical ventilators act in an open-loop manner, but some systems for partial ventilatory support offer dynamical control of the mechanical ventilation through proportional assist ventilation or neurally adjusted ventilatory assist (Yonis et al., 2015). These systems aim to amplify the patient's native intrinsic respiratory effort. The practicality of these designs is limited by the need for non-invasive and reliable measures of the mechanical characteristics of the respiratory system and need for a reliable positioning of an instrumented transtracheal catheter to obtain reliable measure of the diaphragm electrical activity (Navalesi and Costa, 2003; Terzi et al., 2012).

An alternative approach to mechanical ventilation uses trains of electrical pulses to elicit contractions of the paralyzed (or paretic) ventilatory musculature (DiMarco, 2009; Madsen, 2012; Ho et al., 2014). In many individuals, this approach has been used to achieve suitable ventilation, but the performance of existing assistive ventilatory control system technology is limited. Open-loop ventilatory control systems require iterative manual tuning by a practitioner of parameters of a fixed stimulation pattern. Open-loop systems do not have the ability to adjust these parameters if manual tuning does not produce a stimulation pattern that adequately meets metabolic demands. Furthermore, muscle fatigue and changes in electrode response properties can alter the degree of ventilation produced by an open-loop stimulation, which can also result in an unmet respiratory need. In other systems that use electrical stimulation of paralyzed muscles, traditional feedback controllers (e.g., Abbas and Chizeck, 1991; Schearer et al., 2014) and adaptive approaches (e.g., Abbas and Chizeck, 1995; Riess and Abbas, 2001; Fairchild et al., 2010) have been able to achieve high quality control of limb movements. For ventilatory assistance, a closed-loop adaptive control system that uses electrical stimulation could overcome the drawbacks of open-loop control and offer improved quality of life to individuals with paralysis that impairs ventilation.

Neural networks in the brain modulate the drive to the respiratory muscles to meet metabolic demands under diverse conditions. The spatially distributed pontine–medullary respiratory network activates the spinal motor neurons that innervate the respiratory muscles (Richter, 1996; Smith et al., 2009). The activity of the respiratory network is continually adjusted by two major types of feedback: mechanical feedback (lung volume mechanoreceptors) and chemical feedback (levels of CO_2_ and O_2_ in the blood and brain tissue chemical receptors). This activity can be modulated by higher brain centers (voluntary control, posture, emotion, etc.). Therefore, the respiratory network activity is sensitive to various stimuli to drive breathing and non-breathing behaviors (vocalization, eating, straining, airway protection reflexes, e.g., coughing, sneezing, or swallowing, etc.). The respiratory network has a robust yet highly flexible network organization that can permit multiple state-dependent modes of operation (Smith et al., 2009). The neural network can be computationally modeled as a network of spiking neurons. Spiking neural network (SNN) paradigms have gained significant attention in the past decades (Maass, 1997) and have inspired many hardware control systems used for robot control algorithms (Arena et al., 2010; Ayers et al., 2010; Nichols et al., 2013), classifier devices (Hsieh and Tang, 2012), new algorithms (Lagorce et al., 2015), or assistive devices for therapy or rehabilitation (Jung et al., 2001; Vogelstein et al., 2008; Sun et al., 2011; Ambroise et al., 2013). Some closed-loop ventilatory control systems have been proposed (Sharshar et al., 2003; Beck et al., 2007) but none have used a bio-inspired SNN control system.

**Figure 1:**
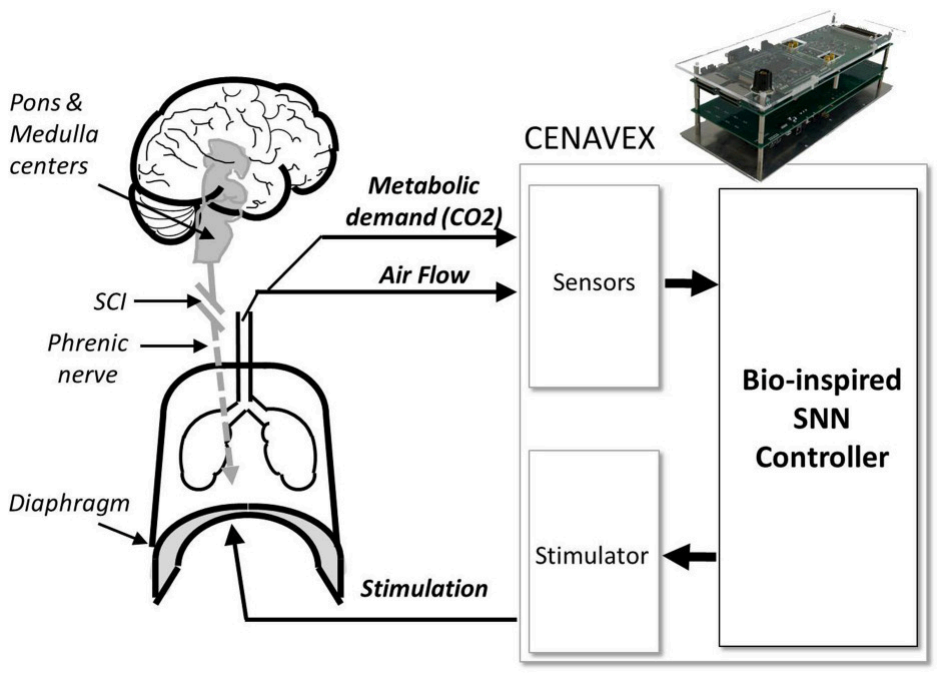
**Closed-loop injured respiratory system with the system Computation-Enabled Adaptive Ventilatory Control System (CENAVEX)**. In the context of spinal cord injury (SCI) or any disruption between the brain and the respiratory muscles, a hardware closed-loop system could provide a complimentary stimulation to the main respiratory muscles (e.g., the diaphragm) for efficient ventilation. A new bio-inspired spiking neural network (SNN) controller drives electric stimulation according to the metabolic status and demands through various physiological feedback signals (air flow, metabolic demand as measured by CO_2_ amount).

In this paper, we address the need for an adaptive ventilatory control system with a new concept: a bio-inspired SNN controller (Figure 1). If the native diaphragmatic drive is totally or partially interrupted by SCI, then ventilation may not be sufficient. A closed-loop system can provide stimulation that adapts in response to measures of physiological variables. Our innovative closed-loop architecture for ventilation assistance is inspired by the pontine–medullary respiratory network to benefit from its robustness and flexibility. The neural network controller is implemented on an FPGA (Field Programmable Gate Array), which is a general approach that has been used since the early 1990s (Cox and Blanz, 1992). It is a fully reconfigurable digital integrated circuit that has low demands on power and computational resources. To achieve high computational efficiency, our controller uses fixed-point computation and implements low computational cost neuron and synapse models. Our design is inspired by the biological circuits but it is not intended to emulate a precise model at the network or cellular level. Our multidisciplinary collaboration has produced a hardware-in-the-loop real-time (HL) simulation platform that uses a computational model of rat ventilation and a fixed-point SNN controller model. The HL simulation was used to validate the bio-inspired SNN controller prior to its implementation in hardware and use in animal experiments.

We first assess the feasibility of implementing a bio-inspired SNN controller in digital hardware. Then, we develop a design methodology for a closed-loop ventilatory control system using our HL computational model and its FPGA implementation. We describe the models (both mathematical and animal) as well as their implementation and use in a HL simulated experiment. Finally, we provide proof-of-concept HL computational results that illustrate ability of the system to adapt the stimulation parameters to meet metabolic demands.

## Materials and methods

### Bio-inspired controller architectures

The neural networks underlying respiratory rhythm and pattern generation and control of ventilation have been studied extensively. Based on these investigations a pontine–medullary respiratory network model has been proposed for control of ventilation (Smith et al., 2000, 2009) that includes a tonic drive network in the pons, the retrotrapezoid nucleus/parafacial respiratory group, and in raphe nuclei; a respiratory rhythm initiating and generating core in the Bötzinger and Pre-Bötzinger regions of the medulla; and follower rostral and caudal ventral respiratory neural network groups. In this model, the core network generates the respiratory rhythm and shapes inspiratory and expiratory motor patterns of the follower ventral respiratory group neurons. The latter contain the pre-motor neurons projecting to the phrenic motoneurons and bulbospinal expiratory neurons for the control of respiratory muscles. The respiratory neural populations are identified by their spiking discharge patterns and by their possible main contribution in ventilatory control (Richter, 1996; Nuding et al., 2009; Smith et al., 2009; Mellen and Thoby-Brisson, 2012; Richter and Smith, 2014). Computational scientists have proposed network models to mimic both the neural population activity patterns and specific modulation of the respiratory network (Lindsey et al., 2012; Molkov et al., 2014; Toporikova et al., 2015).

**Figure 2:**
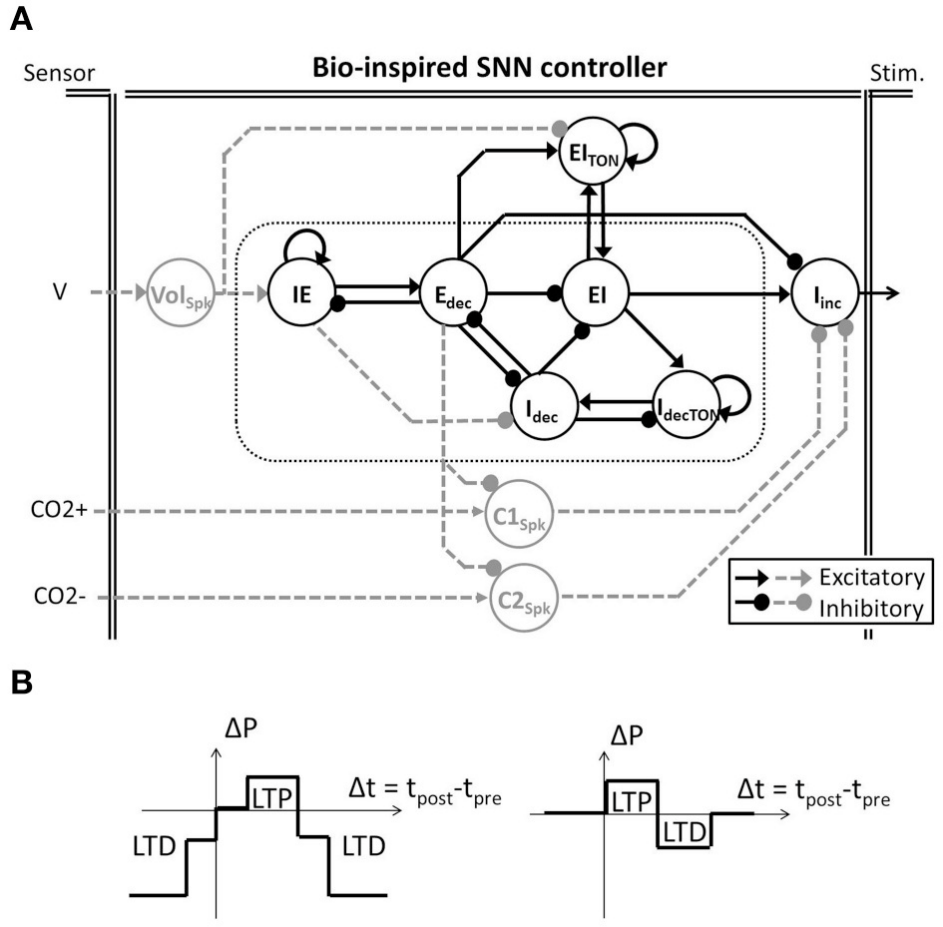
**Bio-inspired Spiking Neural Network (SNN) for ventilatory control systems. (A)** The bio-inpired controller is used in open and closed-loop paradigms (solely black architecture and black and dashed-gray architecture, respectively). Follower I_inc_ neuronal activity triggers and shapes the electrical stimulation accordingly with the core activities (in doted box). In the closed-loop system, gray tonic neurons activities modulate the core behavior accordingly with the sensor measures (Volume V, CO_2_ increase and decrease CO_2+_ and CO_2−_). Tonic neuron EI_*TON*_ is used in both open and closed-loop paradigms to modulate the EI core neuron. **(B)** Two modulations of SNN connection strength (ΔP) rely on SNN learning rules (left and right, respectively). Simplified spike-timing-dependent plasticity includes Long-Term Potentiation (LTP) and Long-Term Depression (LTD).

The bio-inspired SNN controller is based on experimental neuroscience and computational studies of the pontine–medullary respiratory network at a high level (i.e., the architecture) as well as at a low level (i.e., the population activity). We have developed two generations of architecture: an open-loop system (solely black architecture in Figure 2A) and a closed-loop system (both black and gray architecture in Figure 2A). In the network, each SNN controller neuron is inspired by the behavior of one population of the respiratory network. The basic organization includes three sub-networks: the FPGA core network defines the stimulation dynamics for each breath, the FPGA tonic network controls the core network, and the FPGA follower network converts the core network spiking activity into a stimulation signal to the diaphragm. The SNN controller's core and follower neurons are used in both open and closed-loop architectures. Neuron names in the SNN refer to their discharge pattern (*dec* for decreasing, *inc* for increasing) during a respiratory phase (E for expiration, I for inspiration): decreasing activity pattern expiration (E_dec_ neuron), spiking activity during the inspiration-expiration transition (IE neuron), decreasing activity pattern inspiration (I_dec_ neuron), spiking activity during the expiration-inspiration transition (EI neuron), and increasing pattern inspiration (I_inc_ neuron). Other neurons enable reproduction of specific features of the dynamics of the biological network (e.g., additional triggering neurons such as I_decTON_ and EI_TON_ implement excitatory neuron's dynamics). Finally, additional neurons modulate in real-time the bio-inspired SNN controller behavior using sensor information: Vol_Spk_ directly influences volume; C1_Spk_ and C2_Spk_ increase and decrease CO_2_, respectively. CO_2_ was chosen as the primary chemical controller for metabolic demand as end-tidal CO_2_ which reflects the partial pressure of arterial CO_2_ is measureable *in vivo* and provides a very strong influence to respiratory drive.

The bio-inspired SNN controller architecture reflects the respiratory rhythm and pattern generation network architectures presented by Richter and Smith (Richter, 1996; Smith et al., 2009) while being adapted to high density and low power consumption constraints of implanted technology. The Richter and Smith architectures mostly describe the same populations with little variation, such as the core population late-I in Richter (1996) is not explicitly represented in Smith et al. (2009). One common description is the network formed by reciprocal inhibitory connections between two neurons—called the primary inhibitory oscillator. The coupled I_dec_ and E_dec_ neurons mimic the so-called early-I and post-inspiration populations (or PI or post-I).

It should be noted that the SNN architecture does not possess intrinsic pacemakers as in the biological network. The FPGA controller uses a self-excitation connection to mimic intrinsic rhythmogenic capability: the I_decTON_ neuron triggers the I_dec_; the IE neuron triggers the E_dec_ neuron; and the EI_TON_ triggers the EI neuron. Another example is the connections between the core and follower populations. The follower population could either be inhibited by most of the core population (Richter, 1996) or excited by one core population (pre-I/I in Smith et al., 2009) but in both cases it is an excitatory follower population. In the FPGA controller, a self-excitatory connection on the follower I_inc_ neuron is implemented. This self-excitatory connection was already presented in Richter (1996). The EI neuron triggers I_inc_, as suggested by the short activation of the pre-I population prior to the follower activity in Richter (1996). Similarly, the E_dec_ neuron inhibits the follower neuron I_inc_ as suggested by the inhibition of PI neuron on the follower neuron in Richter (1996).

The closed-loop architecture adds three tonic neurons to the open-loop architecture: Vol_spk_, C1_spk_, and C2_spk_ (gray in Figure 2A). Tonic neurons encode signals measured by sensors into spike patterns. The tonic neuron activities can update the strength of specific connections in the bio-inspired SNN controller at each computation step. There is scarce information on the network architecture and the activity of biological tonic populations., Spike-timing-dependent plasticity (STDP) as a Hebbian synaptic learning rule has been identified in biological neurons (Markram et al., 1997; Bi and Poo, 1998). It has been demonstrated in various neural circuits over a wide spectrum of species, from insects to humans (Caporale and Dan, 2008), and various types of STDP have been identified (Abbott and Nelson, 2000). We chose to use simplified STDP learning rules (Figure 2B) to modulate connections according to breathing rate and depth as well as for injury level and metabolic demands: P(f), P(a), and P(inj). While the open-loop controller is triggered by an external excitatory stimulation (not shown in Figure 2A), the closed-loop controller is triggered by the tonic neuron Vol_spk_ activity.

The electrical drive pattern reflects the follower neuron I_inc_ on-going activity. Neuroscience and computational studies usually refer to the neuronal population activity as integrated population activity (population spike frequency histograms) or cycle-triggered histograms (average firing rates of the neurons during the respiratory cycle) reported as spikes per second per neuron (Nuding et al., 2009; Smith et al., 2009). The stimulation ratio is the number of spikes out of 20 measured over the last 20 computation steps (i.e., 10 ms). It represents the activity of each neuron from the bio-inspired SNN. The stimulation ratio is the proportion of maximum activation (stimulus) of the respiratory muscle, 0–1 (in this case, 0 spikes = 0, 10 spikes = 0.5, and 20 spikes = 1, etc.).

### Spiking neural network model

The low complexity leaky integrate-and-fire (LIF) neuron formalism was chosen for its minimal computational demand while maintaining key timing properties of biological neurons. The neuron is modeled as a leaky integrator of its total current from all synapses *I*_tot_*(t)*, defined in Equation (1):

(1) $$\begin{array}{r} \tau_{m}\frac{dv_{m}\left(t\right)}{dt}=-v_{m}\left(t\right)+R_{m}I_{tot}\left(t\right) \end{array}$$

where τ_*m*_ is the membrane time constant, *v*_*m*_*(t)* the displacement of neuron voltage from the resting potential, and *R*_*m*_ the input resistance. The LIF neuron fires a spike when the variable *v*_*m*_*(t)* reaches a fixed threshold. An absolute refractory period forbids the neuron to fire a spike within a fixed period of time after each spike.

The kinetic model for SNN connectivity is an adaptation of the alpha model presented by Destexhe et al. (Destexhe et al., 1994). This kinetic model requires a single multiplier component. It eases the implementation of various kinetics and it “accounts for saturation and summation of multiple synaptic events, obviating the need for event queuing” (Destexhe et al., 1994). The kinetic model is based on a fraction of bound receptors *r(t)*. Destexhe et al. approximate kinetics by two expressions, described in Equations (2) and (3):

1. During a Spike (t0 < t = < t1):
  (2) $$\begin{array}{lll} r\left(t-t0\right) & = & \;r_{\infty }+\left(r\left(t0\right)-\;r_{\infty }\right)\text{ exp}\left(-\frac{t-t0}{\tau_{r}}\right)\;\\ \text{with }r_{\infty } & = & \frac{\alpha }{\alpha +\beta }\&\tau_{r}\;=\;\frac{1}{\alpha +\beta } \end{array}$$
2. After a Spike (t > t1):
  (3) $$\begin{array}{l} r\left(t-t1\right)\;=\;r\left(t1\right)\text{exp}\left(-\beta \left(t-t1\right)\right) \end{array}$$

where *r*_∞_ is the maximum fraction of bound receptors, τ_*r*_ and 1/β are time constants of the two phases of *r(t)* (i.e., during and after spike), and α and β are the forward and backward rate constants for transmitter binding (i.e., how fast or slow the kinetic model is). Unlike the Hodgkin and Huxley conductance model (Hodgkin and Huxley, 1952), α and β in this model are not time-dependent.

The total current from all synapses *I*_*tot*_(*t*) is the sum of synaptic currents *I*_*S*_*(t)*. Each synaptic current is taken to be proportional to the fraction of bound receptors *r(t)*, defined in Equation (4):

(4) $$\begin{array}{l} I_{S}\left(t\right)=A_{SE}\;r\left(t\right) \end{array}$$

where *A*_*SE*_ is the absolute synaptic strength, which can be exhibited only by activating all bound receptors. Nichols et al. (2013) used a similar approach to compute synaptic current to simulate a biologically inspired SNN for robot motion control. They used an alternative kinetic model with three interdependent derivative equations (Tsodyks et al., 1998). Although their system was easily implemented in software for simulation, it was not implemented in hardware. The *R*_*m*_ *I*_*tot*_*(t)* variable, equivalent to the Total Post-synaptic Potential (TPP), is defined in Equation (5):

(5) $$\begin{array}{l} R_{m}I_{tot}\left(t\right)=\underset{i}{\sum }P_{i}\;r_{i}\left(t\right) \end{array}$$

where *P* is the absolute synaptic strength potential as determined by the multiplication of *A*_*SE*_ by *R*_*m*_—the input resistance of the neuron. The input resistance *R*_*m*_ is fixed in this model; *A*_*SE*_ could be modified by plasticity rules. Combining both variables into one, *P*, avoids the use of a multiplier component. Finally, absolute synaptic strength potential is used as the connection strength in the bio-inspired SNN network.

To summarize, the neural network model is defined by three processes. The “Kinetic” process updates the fraction of bound receptors *r(t)* according to defined kinetic model. The “TPP” process computes each neuron *R*_*m*_ *I*_*tot*_*(t)* variable. The “V_*m*_ & Spike” process updates the *V*_*m*_*(t)* variable, the refractory period status, and may fire a new spike according to the defined LIF neuron model.

### FPGA implementation (open-loop)

The bio-inspired SNN controller was described in Very High Speed Integrated Circuits Hardware Description Language (VHDL) to define how to use and connect the hardware components available on the FPGA. The SNN controller was implemented on a Xilinx Spartan-6 FPGA (XC6SLX150: 4 Mbit full speed block RAM, 180 embedded multipliers, 92 k 6-input look-up tables and 184 kb distributed memory), hosted on a custom board, presented elsewhere (Zbrzeski et al., 2015). We used UART communication for storage and display of real-time variable data from the SNN.

**Figure 3:**
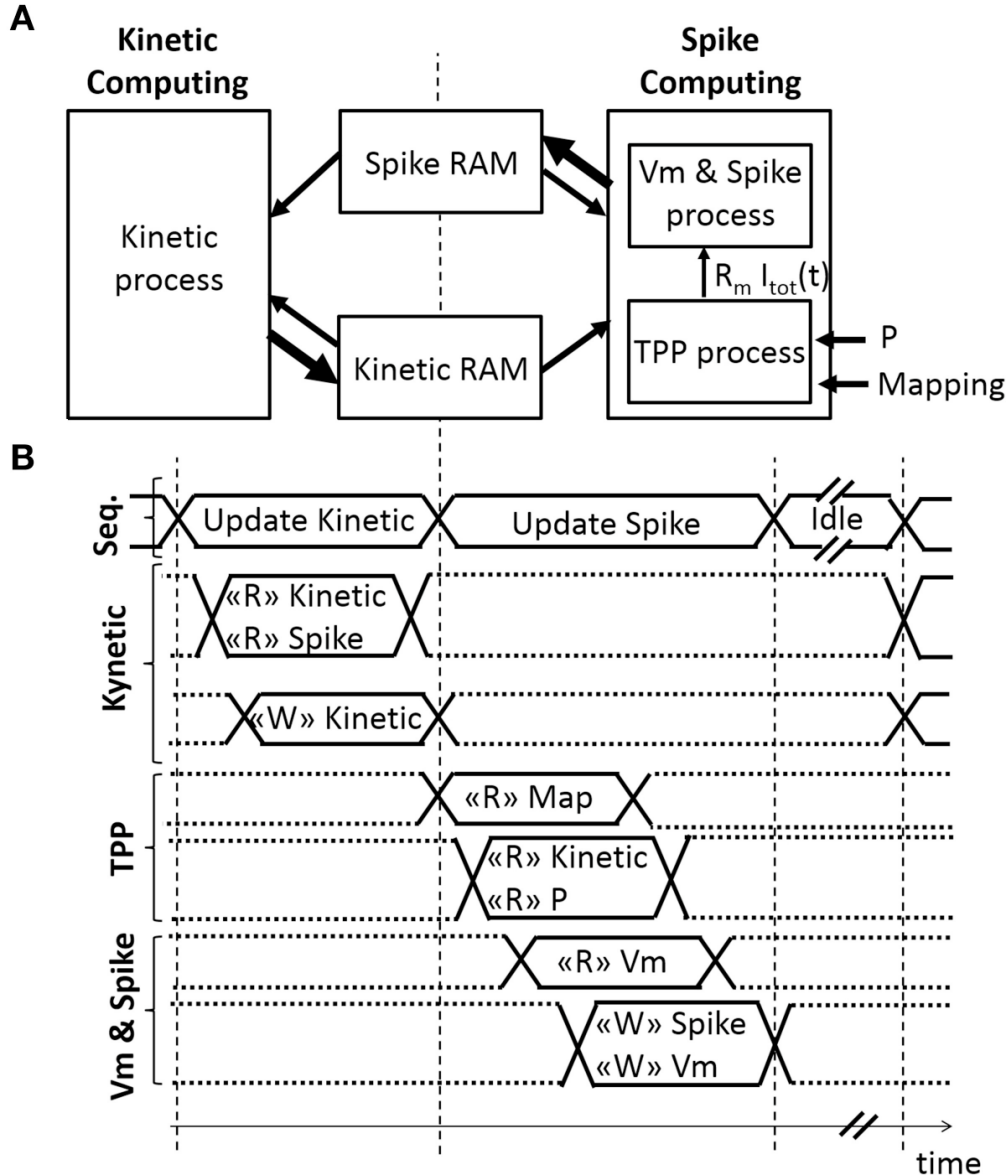
**Architecture of the Spiking Neural Network (SNN) implemented on an FPGA. (A)** Spatial and **(B)** temporal SNN representations, with *r(t)* the fraction of bound receptors stored in the Kinetic RAM, *P* the absolute synaptic strength potential, *R*_*m*_ *I*_*tot*_*(t)* the Total Post-synaptic Potential (TPP), and *Vm* the displacement of neuron voltage from the resting potential stored in the Spike RAM as well as the spiking activity of the associated neuron. Small or bold arrows represent, respectively, a computing process reading “R” or writing “W” in a Random-Access Memory (RAM). The SNN sequencer (Seq.) manages two computing phases, followed by an idle phase. The first computing phase updates each kinetic variable *r(t)* associated with one pre-neuron. The second computing phase updates the spike status for each neuron by computing *R*_*m*_ *I*_*tot*_*(t)* variable with the TPP process, followed by computing *Vm*, the refractory period, and the spike status with the “Vm & Spike.” The SNN connectivity is stored in mapping RAM.

A sequencer coordinates two computing phases of the SNN that update and share two dynamical variables, *r(t)* and *V*_*m*_*(t)* (Figures 3A,B). Both computing phases rely on the previously defined models, taking advantage of their simplicity and their computational efficiency. Then, the sequencer controls a third idle phase to refresh the variables at a defined frequency (in order to achieve biological real-time). In this application, real-time is defined by the controller's ability to elicit diaphragm contraction with on-going real-time updates of the stimulation parameters. The stimulation frequency range is between 75 Hz (typical) and 500 Hz in open-loop stimulation on a rat phrenic nerve or diaphragm (injured or not) (Mantilla et al., 2010; Kowalski et al., 2013). Stimulation frequency is lower for larger animals (e.g., dog, 10–50 Hz) (Walter et al., 2011). The bio-inspired controller updates the SNN behavior and variables at 2 kHz, i.e., the computation time-step *dt* is 0.5 ms. It does not depend on the number of neurons and it ensures accurate control of stimulation pattern.

The first phase is the Kinetic computing. The “Kinetic” process is the conversion over discrete *dt* time-steps of the fraction of bound receptors *r(t)* from the model defined in Equations (6)–(8) using forward Euler integration.

1. During a spike (t0 < t = < t1)
  (6) $$\begin{array}{l} r\left[t+1\right]=A\;r\left[t\right]+B \end{array}$$
2. After a spike (t > t1)
  (7) $$\begin{array}{l} r\left[t+1\right]=C\;r\left[t\right] \end{array}$$

where A, B, C are constants defined as.

(8) $$\begin{array}{l} A=dt\;\alpha \;B=1-dt\;\left(\alpha +\beta \right)\;C=1-\left(\beta ∕dt\right) \end{array}$$

Table 1 presents five sets of kinetic parameters associated with one neuron. Each set consists of 4 parameters: the maximum fraction of bound receptors (*r*_∞_), the rising exponential time constant (τ_*r*_), and the forward (α) and backward (β) rate constants for transmitter binding. Each kinetic parameter set determines the values of constants (*A, B*, and *C*) that are implemented using 14-bit unsigned integers to streamline real-time computation. A 3-stage pipeline is used to update all five kinetic parameters connected to one neuron output. One multiplier is used per kinetic parameter. It takes 519 clock cycles to update 512^*^5 fractions of bound receptors *r(t)*, saved in block RAM as 0.18 unsigned vectors (i.e., rational values between 0 and 1 coded on 18 bits).

**Table 1:** **Available kinetics parameter sets**.

**Kinetic set #**	**r_∞_**	**τ_*r*_ (ms)**	**α (ms^−1^)**	**β (ms^−1^)**	**A**	**B**	**C**
5	0.90	1.0	900	100	0.4500	0.5000	0.9500
4	0.90	2.0	450	50	0.2250	0.7500	0.9750
3	0.92	4.0	230	20	0.1150	0.8750	0.9900
2	0.92	8.0	115	10	0.0575	0.9375	0.9950
1	0.92	15.9	58	5	0.0290	0.9685	0.9975

*Each row presents a pre-defined parameter set that is available for selection when implementing the controller. The α and β are the forward and backward rate constants for transmitter binding. r_8_ is the maximum fraction of bound receptors. τ_r_ is the time constant of the increasing exponential characterizing the r(t) behavior during a spike. A, B, C are the unsigned 14-bit constants used for implementation on the FPGA*.

The second phase consists of computing spikes. It is the conversion over discrete *dt* time-steps of the LIF model using forward Euler integration, defined in Equation (9). After a spike, a neuron has a minimum refractory time of one time-step during which membrane potential is set at its resting potential (in this case, −70 mV).

(9) $$\begin{array}{lll} v_{m}\left[t+1\right] & = & v_{m}\left[t\right]+\frac{dt}{\tau_{m}}\left(v_{rest}-v_{m}\left[t\right]+R_{m}I_{tot}\left[t\right]\right)\\ \text{with }R_{m}I_{tot}\left[t\right] & = & \sum P\;r\left[t\right] \end{array}$$

The ratio *dt/*τ_*m*_ is set at 8 to use a shift operation instead of a multiplication. The “TPP” process updates the *R*_*m*_ *I*_*tot*_[*t*] signal, as illustrated in Figures 3A,B. The *R*_*m*_ *I*_*tot*_[*t*] signal is connected to the “V_*m*_ & Spike” process. The implemented architecture does not include the plasticity mechanism, but the block RAM accesses are already available for such a mechanism. The “TPP” and “V_*m*_ & Spike” processes each use a 3-stage pipeline to update all neurons. Four multipliers are used in the “TPP” process. It takes 1036 clock cycles to update 512 neurons during this second phase.

The SNN architecture can construct up to an 8-to-all 512-neuron network. Each connection is modeled by 1 among the 5 possible kinetic connections of Table 1. The implemented network has a low connectivity density. The implemented SNN controller uses the open-loop architecture (Figure 2A). Table 2 presents the open-loop SNN synaptic parameters.

**Table 2:** **Connectivity between the tonic and core populations of the open-loop SNN controller implemented on the FPGA**.

	**Post-I**		**Early-I**		**PreI/I**		**-**
	**Edec_TON_**	**Edec_OUT_**	**Idec_TON_**	**Idec_OUT_**	**EI_TON_**	**EI_OUT_**	**ON**
***i***	**K_i,1;_ P_i,1_**	**K_i,2;_ P_i,2_**	**K_i,3;_ P_i,3_**	**K_i,4;_ P_i,4_**	**K_i,5;_ P_i,5_**	**K_i,6;_ P_i,6_**	**K_i,7;_ P_i,7_**
1: Edec_TON_	3; +600	1; +5000	–	–	–	–	–
2: Edec_OUT_	1; −2200	–	–	4; −250	4; +8000	4; −500	–
3: Idec_TON_	–	–	3; +40	4; +500	–	–	–
4: Idec_OUT_	–	1; −1500	3; −5000	–	–	4; −350	–
5: EI_TON_	–	–	–	–	3; +40	2; +400	–
6: EI_OUT_	–	–	2; +200	–	2; +1280	–	–
7: ON	4; +1280	–	–	–	–	–	–

*Each row corresponds to a pre-synaptic neuron i; each column corresponds to a post-synaptic neuron, j. For each pair of connected neurons, the first value indicates the kinetic connection K_i,j_ between neurons i and j; the second value indicates the absolute synaptic strength potential P_i,j_. Note that the ON neuron is used to automatically start the SNN controller after its initializations when used in open-loop mode*.

### Hardware-in-the-loop real-time computational model

**Figure 4:**
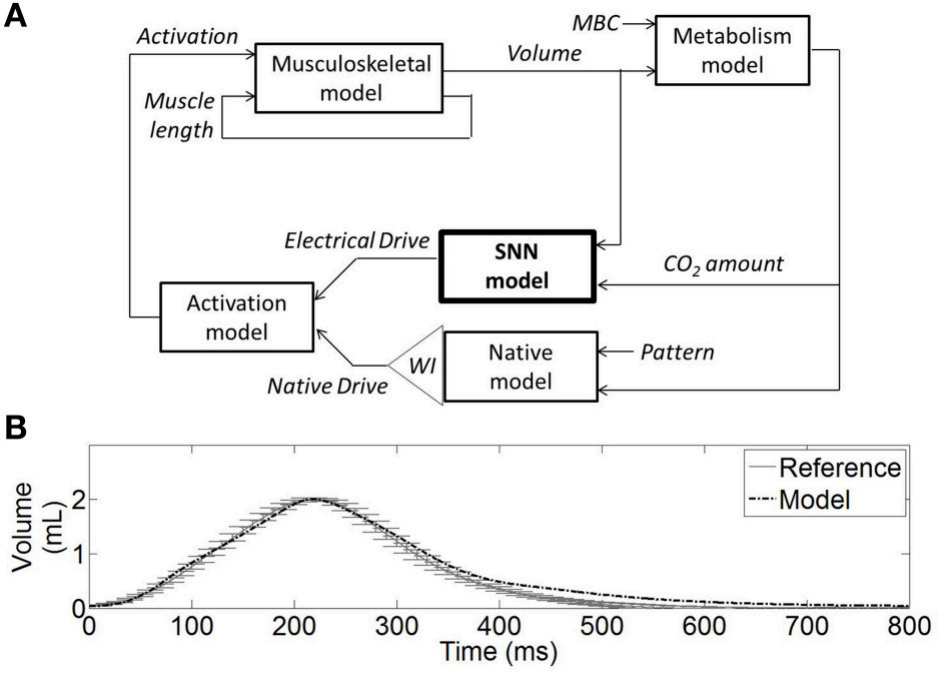
**Hardware-in-the-loop (HL) real-time computational model. (A)** Diagram of the HL computational model including the SNN controller model (bold) and the rat computational model. The SNN controller model (electrical stimulation) and the native model drives (phrenic nerve) are combined in an activation signal (normalized) to stimulate the muscle model. The musculoskeletal model provides the dynamic volume. The metabolism model provides the CO_2_ amount in the body, considering a fixed reference CO_2_ production (or metabolic demands parameter MBC). The weighted injury factor WI can attenuate the native drive to reflect the impact of spinal cord injury on ventilation. The native drive pattern was defined to mimic experimental data (see **B**). The native model includes a dynamical respiratory rate mechanism: it modulates the native drive pace to reach a constant CO_2_ amount on a breath-by-breath basis. **(B)** The non-injured native activation elicits a volume (Model) that is similar to experimental rat volume (Reference).

The bio-inspired SNN controller can provide complementary diaphragmatic electrical stimulation to the native phrenic stimulation. The closed-loop version of the SNN controller adapts the stimulation (timing, amplitude) to meet the metabolic demands by adapting the depth and frequency of breathing. As a precursor to animal experiments, the SNN controller was evaluated in a computational model. Figure 4A illustrates a hardware-in-the-loop real-time (HL) computational model using a rat computational model and a fixed-point FPGA SNN controller model. The HL model maintains real-time processing capability (0.5 ms).

The computational model of rat ventilation is a real-time dynamical model with the ability to simulate rat ventilation for various breathing states. The bio-computational model includes a native drive model, an activation model (a linear summation of native and stimulated activation, a simplified first approximation), a musculoskeletal model, and a metabolism model. The model was developed to reproduce the phrenic native drive of an injured/non-injured animal, the combined muscle activation drive, eupneic respiratory volume, and a residual CO_2_ quantity in the model.

The musculoskeletal model was scaled to fit the eupneic respiratory output of a non-injured rat. This model was developed from a human computational model in Simulink/SimMechanics implementing the physiologically realistic muscle model from MSMS (Hillen and Jung, 2014). Proportional muscle activation (0–1, proportion of spikes out of 20) as provided by the SNN was transformed into motor unit activation using the intramuscular functional electrical stimulation model from Virtual Muscle (Song et al., 2008) as implemented in MSMS which uses a single motor unit of each type where firing frequency is specified for stimulus intensity using a recruitment order from Singh et al. (2000) for intramuscular stimulation. The lung volume displacement was assumed linearly related to the diaphragm displacement, as illustrated in Richter (1996). This approximation was determined to be acceptable, especially when considering the system in eupneic conditions and when focusing on the volume shape during inspiration phase. Ventilatory compliance was modeled as a damped spring with non-linear stiffness. The non-linear stiffness values for the rat thorax/diaphragm were chosen from experimental data (Young et al., 1992). Damped spring and activation amplitude from the human musculoskeletal model were adjusted such that the rat musculoskeletal model produced lung volume trajectories similar to experimental collected rat data (10 experimental data recordings, as presented in Figure 4B). Native activation was represented as a spaced sawtooth function and defined the pattern of the native model.

Experimental data from one male Sprague Dawley rat (*n* = 1, 360 g) (Siu et al., 2015) was used to determine the desired lung volume waveforms that were then used by the control system in the simulation studies. Data was collected with the approval of the Institutional Animal Care and Use Committee of Florida International University. The rat was maintained under eupneic conditions (21% O_2_, 0% CO_2_, balance N_2_) through the use of a gas mixer (GSM-3, CWE Inc.). The rat was anesthetized via IP delivery of pentobarbital (45 mg/kg) with supplemental isoflurane (0.5–1%). A pulse oximeter monitored SpO_2_, while end-tidal CO_2_ was monitored via a CO_2_ analyzer (Capstar-100, CWE Inc.) throughout the study to monitor and assess animal health. A pneumotachometer (8420A, Hans-Rudolph) collected air flow, which was then processed through a hardware integrator (PI-1000, CWE Inc.) to obtain breath volume. Bipolar stainless steel barb electrodes were inserted into the diaphragm to record electromyography. Flow, volume, end tidal CO_2_, and electromyography measurements were collected at 6 kHz.

The metabolism model was created to dynamically represent the quantity of CO_2_ in the computational model. The metabolism model describes generation of CO_2_ (by mass) in the body and the exhalation of CO_2_ with each breath (as determined by breath volume/rate). Baseline CO_2_ production (MBC0) was set for net-zero increase in CO_2_ in the body during tidal breathing/eupnea. Increases in metabolic demand are represented by increases in CO_2_ production rate. The eupneic computational condition was defined for a respiratory rate (RR) of 56 breaths per minute and a tidal volume of 2 mL. The CO_2_ amount is constant on a breath-by-breath basis. If there is an increased metabolic demand while the rat computational model RR and/or volume are not altered, then the CO_2_ amount will increase on a breath-by-breath basis.

The native model output is the native drive (e.g., phrenic drive) with a fixed amplitude, and fixed or variable respiratory rate. In a biological rat, RR and tidal volume would increase to keep the CO_2_ amount constant. A linear regression defines the relationship between RRs and MBC parameters to provide constant CO_2_ amount. It defines the dynamical RR mechanism. Increased metabolic demand conditions of +11% and +17% are called MBC11 and MBC17. These two increased metabolic demands lead to a RR of 62.31 and 65.75 breaths per minute in the non-injured rat model to maintain constant average CO_2_. Also, the native model was varied to roughly mimic the effect of an incomplete spinal cord injury on ventilation by attenuation of the native drive through a weighted injury factor. In experimental studies, at 1 day post-injury and in the eupneic condition, rat tidal volumes were reported to be between 70 and 90% of the non-injured tidal volumes (Fuller et al., 2006; Navarrete-Opazo et al., 2015). For use in these simulations, we defined 6 pairs of injury level and metabolic demand conditions: 90%-MBC11, 90%-MBC17, 80%-MBC11, 80%-MBC17, 75%-MBC11, 75%-MBC17.

The SNN controller provides a simulated electrical drive. The SNN network was simulated using the computing phases, computing algorithms and pipeline implementations, and hardware limitations (fixed-point dynamic and accuracy), detailed in section FPGA Implementation (Open-Loop). The HL computational model predicts the behavior of the FPGA implementation of the SNN controller.

Computational results for both open and closed-loop SNN controllers are presented. The open-loop SNN controller was simulated with parameters that represent a complete injured rat computational model (native drive: 0%) with normal metabolic demands (MBC0). The closed-loop SNN controller was simulated with parameters that represent an incomplete injured rat computational model (native drive: 90, 80, 75%) with normal and increased metabolic demands (MBC0, MBC11, MBC17). The coefficient of variation (CV) of RR was computed over the last 10 breathing cycles to assess the variation of the RR over time. Constant RR was defined as breathing that exhibited a coefficient of variation of RR less than 4%.

The same SNN architecture was used for all models. The strength synaptic potentials *P(inj)* were set to optimize the efficiency of the bio-inspired controller for each pair of injury level and metabolic demand. *P(a)* and *P(f)* were set only in open-loop configuration, and were adapted by the controller in the closed-loop configuration. Breathing conditions are characterized with respiratory rate using the 10 last breathing cycles, and the related coefficient of variation.

## Results

### Complete injury experiment with open-loop controller

**Figure 5:**
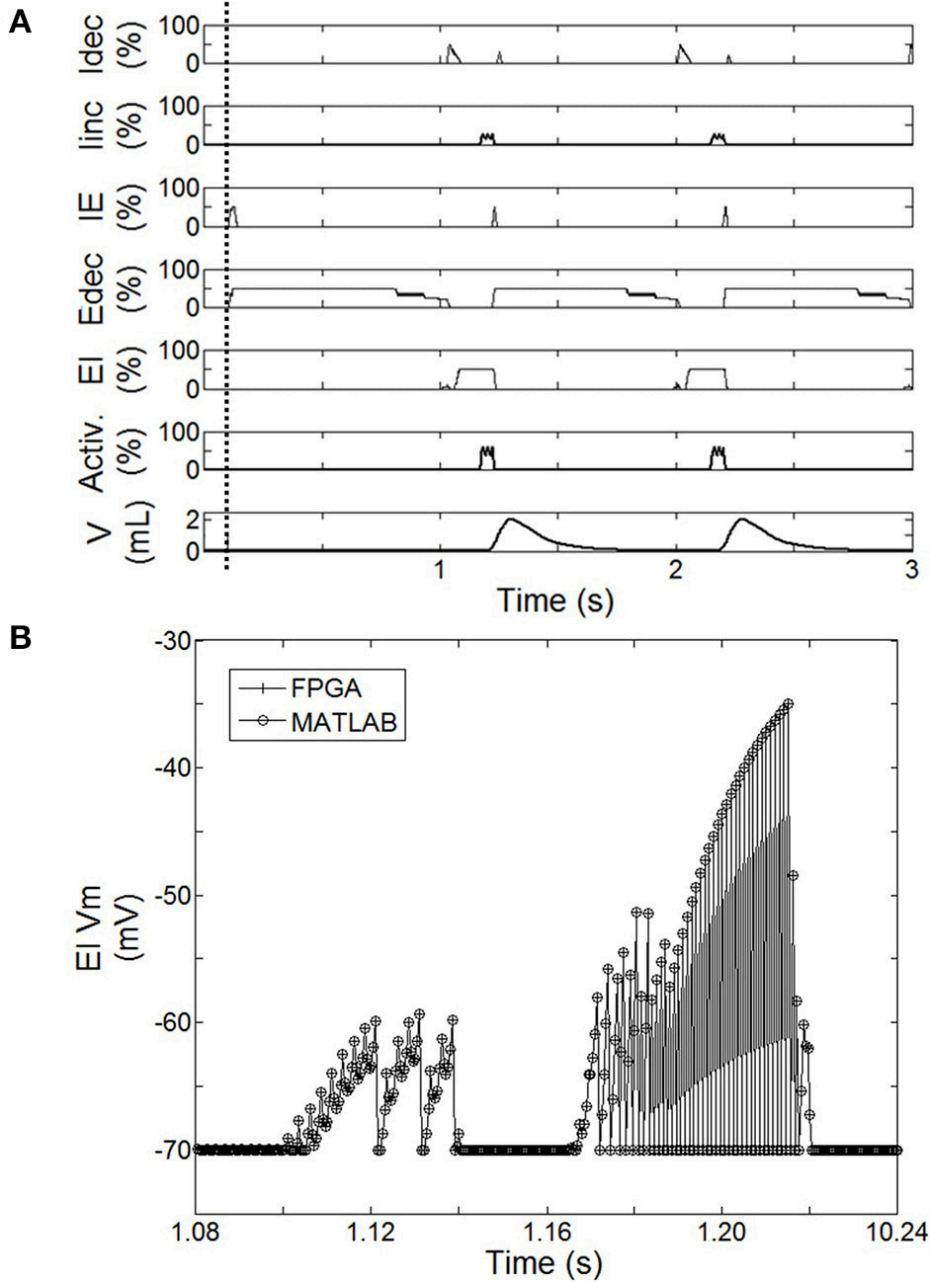
**Behavior of the spiking neural network (SNN) when the system is turned ON (after dashed line). (A)** Averaged spike count on the last 20 computation steps for each SNN neuron (I_dec_, I_inc_, IE, E_dec_, EI). 100% means the neuron has spiked 20 times over the last 20 computation steps. Results are computed using the hardware-in-the-loop (HL) real-time computational model. SNN model uses the first architecture presented Figure 2A with a complete injured model (native drive 0%). Activation drive Activ. and Volume V are reported. **(B)** Illustration of the exact match between FPGA measure and HL computation. Example using the displacement of neuron voltage from the resting potential (Vm) for EI neuron.

The bio-inspired controller described in Section Materials and Methods was used with the complete lesion model (native drive: 0%): diaphragm activation was specified as described in Section Bio-Inspired Controller Architectures. The electrical drive mimics the activity of the follower neuron I_inc_ (Figure 5A). Both frequency and amplitude of the electrical drive are fixed in the open-loop paradigm. The open-loop bio-inspired SNN controller uses 7 neurons and 17 connections with low computational power (e.g., neuron and kinetic simplified behavioral models). Device utilization after synthesis of the SNN controller for the open-loop system is listed in Table 3. The design operates at 124 MHz. Mixed pipeline, parallel structure and shift operations were used to design a network with optimized hardware resource costs.

**Table 3:** **Device utilization (Xilinx Spartan XC6SLX150) for the implementation of the open-loop SNN controller on FPGA**.

**Resource**	**Used**	**Percent utilization (%)**
Slice LUTs	4502	4
LUT-FF pairs	2124	36
Slice registers	3427	1
18Kb RAM/FIFO	19	7
Mults (18 × 18)	9	5

*Results obtained using XST synthesis software from Xilinx Inc. with standard optimization effort on timing, under the ISE 14.7 development environment*.

The activity of this bio-inspired network is similar to experimental reports (Richter, 1996), as reflected in activity patterns of individual populations and in temporal sequencing and coordination across the network (Figure 5A). The coordinated pattern of observed activity is as follows: the I_dec_ activity increases quickly before slightly decreasing, this is followed by a slight then a fast increase of I_inc_ activity; the I_inc_ activity decreases abruptly when the IE activity quickly increases; this triggers E_dec_ activity (and IE activity goes off); then E_dec_ activity increases first quickly then slowly, which turns on EI activity to briefly activate I_dec_ activity; and the activation sequence is repeated. The Matlab SNN computational model was designed to emulate the high level dynamic behavior as well as the exact data processing dynamics of the FPGA component. The HL computational delay is the computation time-step itself; there is no buffering effect at the network level. The MATLAB implementation was used to develop the FPGA implementation, which runs in real-time with sub-microsecond precision. Dynamic behaviors and values are strictly the same between the computational model running on Matlab and its FPGA implementation from the network to intrinsic neuron variables as illustrated in Figure 5B. This observation was verified for all SNN variables: membranes voltages, spike states and fractions of bound receptors of the kinetic model (not shown).

**Figure 6:**
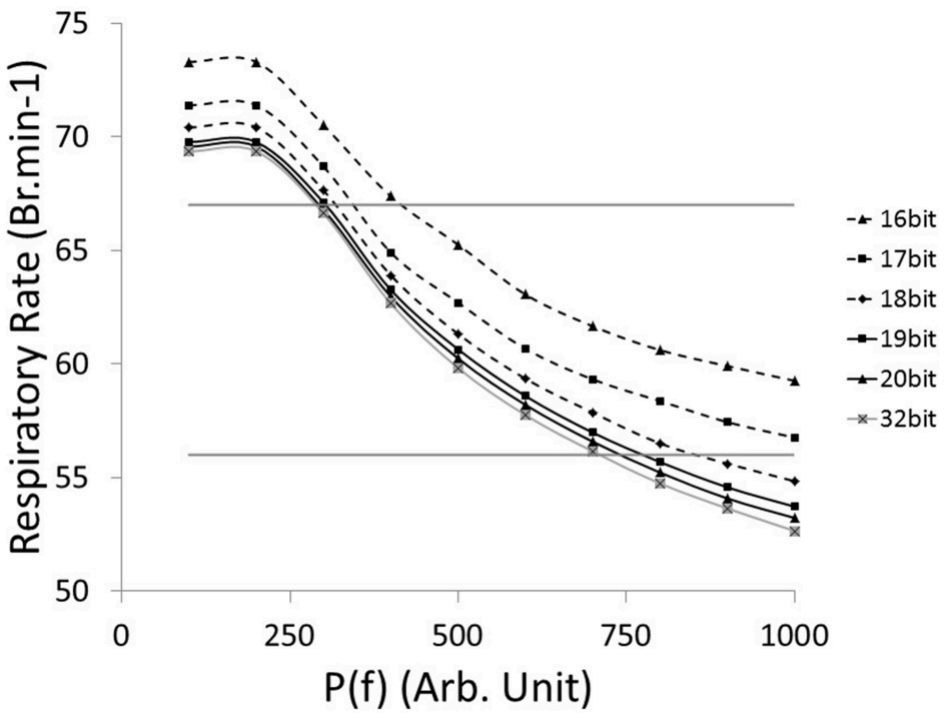
**Computational Respiratory rate (RR) vs. tuned open-loop bio-inspired controller**. The absolute synaptic strength potential between pre-neuron IE and post-neuron E_dec_ is P(f), identified in Figure 2A. Fractions of bound receptors of the kinetic model can have an accuracy ranging from 16 bit to 32 bit. Native drive is 0 to mimic a complete injured model. Horizontal lines identify RR at 56 and 67 Br.min^−1^, targeting range of rat eupneic RR.

The relationship between the accuracy of the fractions of bound receptors and the system's ability to drive a wide range of stimulated respiratory rates is presented in Figure 6. We evaluated the impact of *r(t)* accuracy (number of bits) on the SNN output resolution. The HL computational tool facilitates the identification of the most sensitive absolute synaptic strength potentials, P(f), to the modulation of electrical drive frequency (i.e., the node between pre-neuron IE and post-neuron E_dec_ neurons). The HL computational tool helps to find appropriate tradeoffs between technology resources and application function in a varied respiratory context. All SNN variable accuracies are fixed except for the *r(t)* accuracy (kinetic model). The maximum RR variation between bio-inspired controller with the highest simulated *r(t)* accuracy (32-bit) and lower accuracies (under, at and above 18-bit) are, respectively 4, 2, and 1 Br.min^−1^. Another comparison is set by targeting a range of rat eupneic RRs (e.g., between 56 and 67 Br.min^−1^). The associated range of absolute synaptic strength potentials *P* with high accuracy (32 bit) is from 300 to 700. The bio-inspired controller with *r(t)* accuracy above 18-bit approximately shares the same lower range, and can vary up to 100 with the lowest accuracy. The *r(t)* accuracy has a stronger impact on the higher range, e.g., from 700 (32-bit) to 850 (18-bit). Therefore, using low accuracy could potentially limit the ability of the system to deliver stimulation at a high frequency. Finally, we used 18-bit accuracy for the fractions of bound receptors *r(t)* in the bio-inspired controller: it has shown to be closely equivalent to a 32-bit structure and it minimizes the number of multipliers (limited in this technology to a 18-bit accuracy).

### Incomplete injury experiment with closed-loop controller

**Figure 7:**
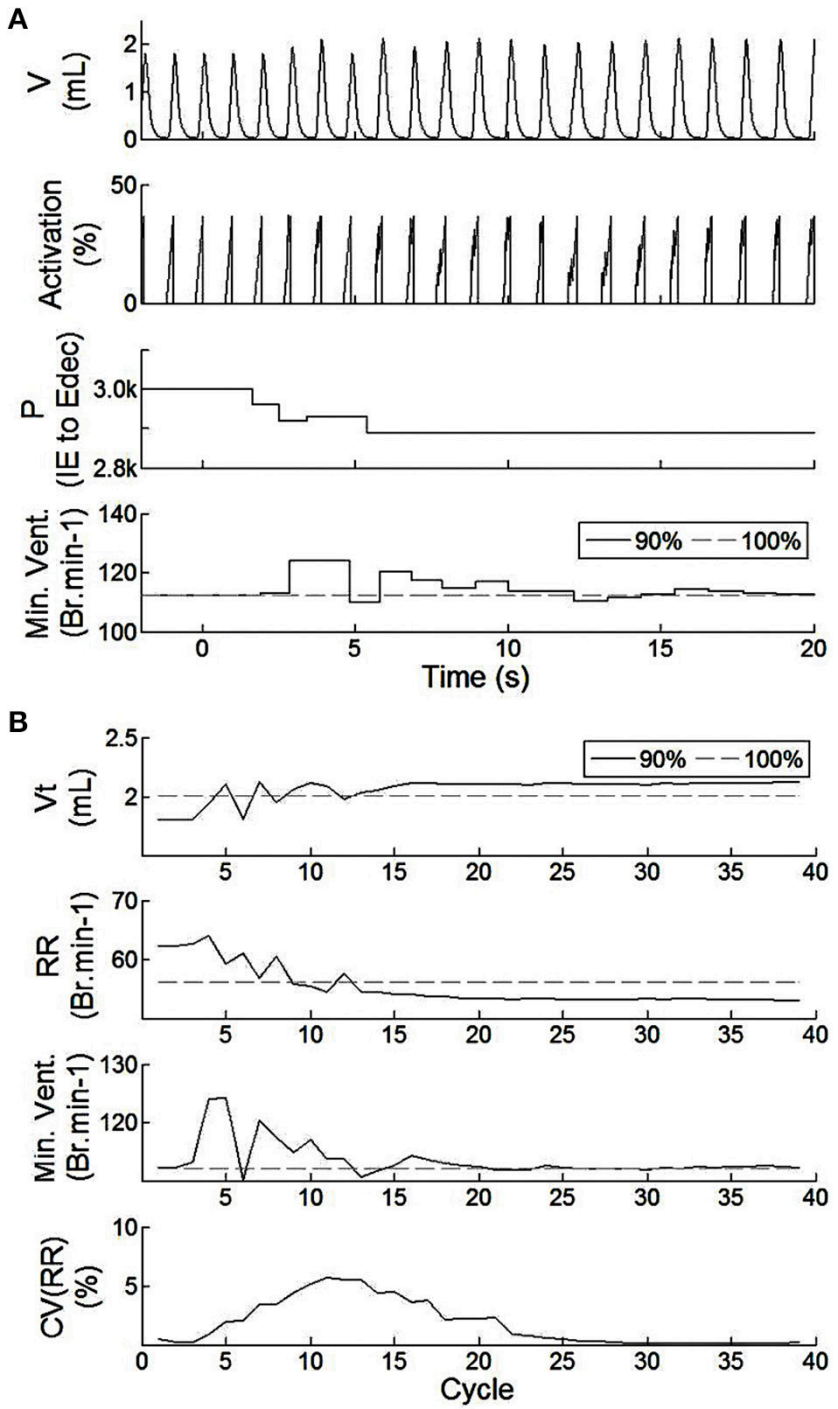
**Effect of the SNN electrical stimulation on the ventilation of an incomplete injured rat illustrated by hardware-in-the-loop real-time computational model in eupnea. (A)** Electrical drive effect on the rat computational breathing over time when the SNN controller was turned ON (at *t* = 0 s). Volume V, Activation drive to the musculoskeletal model, and absolute synaptic strength potential P between the IE and Edec population are presented for incomplete injured model. Minute ventilation (Min.Vent.) is compared between incomplete injured and non-injured models. Associated native drives are 90% and 100%. **(B)** Evolution of respiratory parameters vs. breathing cycle. The SNN controller was turned ON at cycle #1. Tidal volume (Vt), respiratory rate (RR), minute ventilation (Min.Vent.), and coefficient of variation of the RR parameter (over the last 10 cycles) are presented for both incomplete injured and non-injured models.

The closed-loop architecture of bio-inspired SNN controller was designed, developed, and modeled for HL computation. It can dynamically synchronize its electrical drive with the native drive. The electrical drive can modify simulated animal breathing depth as well as respiratory rate. Figure 7 shows the effect of activating the bio-inspired controller in an incomplete injury model (native drive: 90%). Breathing was not optimal prior to activation of the bio-inspired controller; depth of breathing was lower than in the non-injured model. The metabolism model sets a slightly higher breathing rate in the injured than in the non-injured model to keep equivalent minute ventilation: respiration depth was higher in the injured model with bio-inspired controller as compared to the non-injured model. The CV of RR reaches a peak of approximately 5% at cycle #11. The stabilization of the amplitude and frequency of respiration occurs in the 16th breath cycle, about 15 s after activation of the bio-controller while the CV of the respiratory rate parameter drops below the 4% mark. The activation pattern after *t* = 15 s was consistent. The stabilization of P(f) (sensitive to electrical stimulation frequency) has a faster timescale of 4 cycles (*t* = 6 s). The absolute synaptic strength potential, P(a), did not vary much upon activation of the bio-inspired controller (not shown).

**Figure 8:**
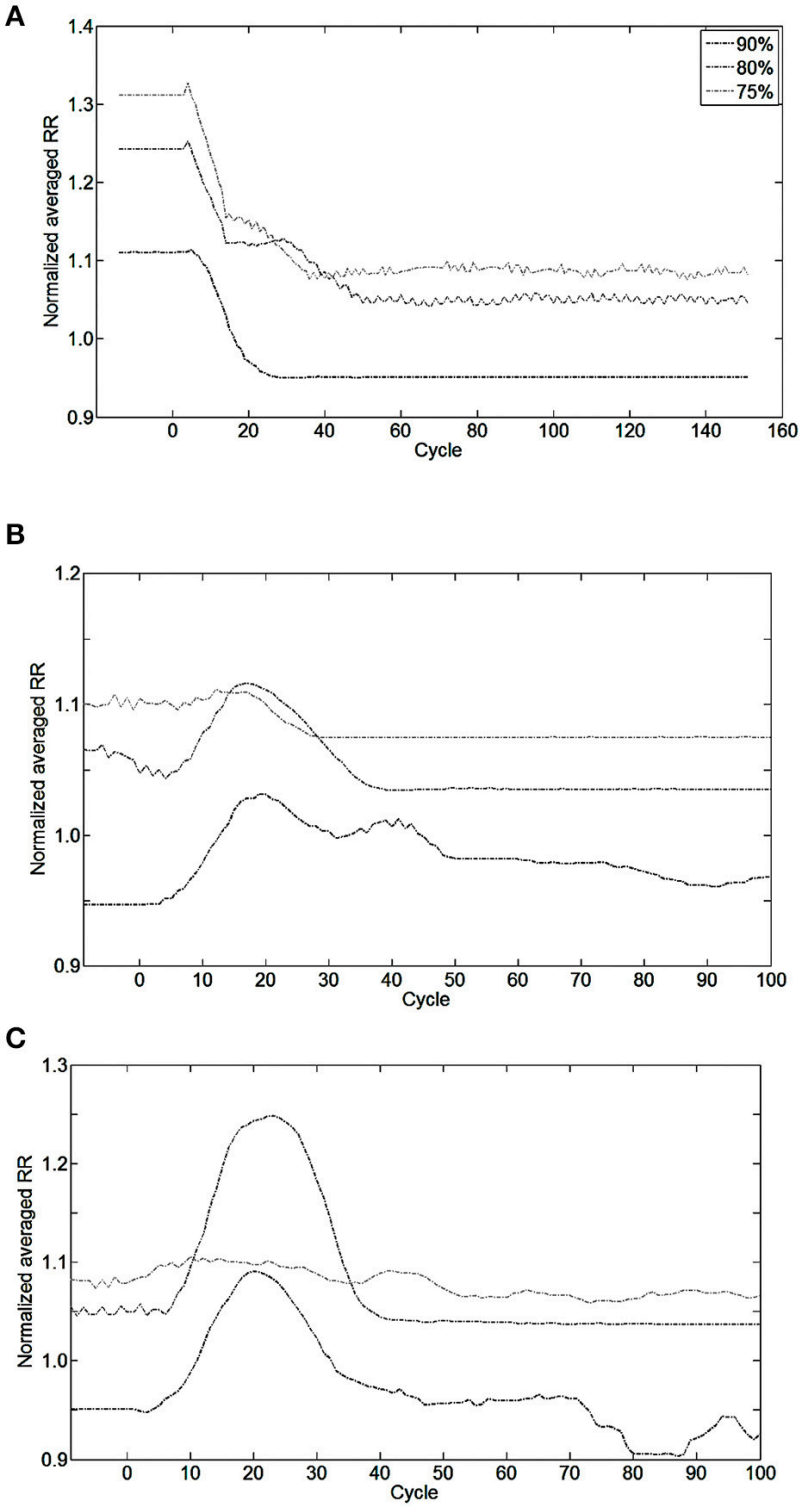
**Effect of bio-inspired controller electrical stimulation on breathing using injured model (native drive: 90, 80, 75%) vs. non-injured model (native drive: 100%)**. The normalized averaged respiratory rate (RR) is the ratio of averaged RR over the last 10 breathing cycles between injured and non-injured models. Normalized averaged RR of 1.0 means the averaged RR is equal between injured and non-injured models. Perturbations start at cycle #0. **(A)** The bio-inspired controller was turned ON at cycle #0 using initialization parameters set for a future metabolic demand increase of 17%. Results of the bio-inspired controller set for a future metabolic demand increase of 11% are not shown for readability. **(B)** Metabolic demand increased from eupneic to +11%, or **(C)** to +17% at cycle #0. Controller was turned ON at cycle #0 in **(B,C)**. At cycle #0, the non-injured RR was set at the final metabolic demand non-injured RR is 56.0, 62.2, 65.7 Br.min^−1^ in, respectively, **(A–C)** results.

The closed-loop system demonstrated robust performance when tested on 3 types of incomplete injury (native drive: 90, 80, 75%) with perturbations induced by activating the bio-inspired controller and by alterations in the rat model (sudden increases in metabolism of 11 and 17%). The bio-inspired controller performed well in all cases by responding to the perturbation. The bio-inspired controller demonstrated adaptability by modifying the electrical drive to assist the incomplete injured model to reach equivalent breath depth and rate as in a non-injured model. The HL computational results with respect to a non-injured model are presented in Figure 8 for all pairs of injury level and metabolic demand and for all perturbations. The absolute synaptic strength potentials P(inj) are more sensitive to the injury level than the metabolic demand increase except for one of them (*P* between Vol_spk_ and IE neurons, not shown). Data quantifying the effectiveness of the bio-inspired controller are presented in Table 4. First, the breath depth and rate were evaluated when the bio-controller was turned ON (Figure 8A). Initially, with the bio-controller OFF, the respiratory rates were all higher when the level of injury was large: respiratory rate increased from 11 to 31% from a non-injured model. Respiratory rate was reduced in all cases when the bio-controller was turned ON: a 4–10% error was measured when compared to the non-injured model but with a 5–23% improvement when compared to incomplete injured models with the bio-inspired controller OFF. The stabilization of the synchronized bio-inspired controller was reached in 14 breathing cycles with high injury models (80 and 75%) vs. 17 cycles with the lowest injury model (90%). The performance after activation of the bio-inspired controller was independent of network parameters for each injury level: e.g., injury at 90% has both a synchronization time and stabilization time of 17 breathing cycles.

**Table 4:** **Delay of the bio-inspired controller to adapt to various perturbations expressed in breathing cycles**.

**Network vs. perturbation**	**OFF/ON**	**+MBC**
90%–MBC11	17 cycles (1.11–0.94)	13 cycles (0.94–0.96)
90%–MBC11	17 cycles (1.11–0.95)	15 cycles (0.95–0.92)
80%–MBC11	14 cycles (1.24–1.06)	13 cycles (1.04–1.03)
80%–MBC17	14 cycles (1.24–1.04)	17 cycles (1.04–1.03)
75%–MBC11	14 cycles (1.31–1.10)	10 cycles (1.10–1.07)
75%–MBC17	14 cycles (1.31–1.08)	13 cycles (1.07–1.06)

*Perturbation is either when the controller is turned ON (OFF/ON) or when the metabolic demand increases (from eupneic to +11% or +17%) while the controller is ON. Injury level is defined by the native drive percentage (90, 80, 75%). One spiking neural network architecture was set with 6 slight variations of few absolute synaptic strength potentials to optimize the efficiency of the bio-inspired controller for each pair of injury level/metabolic demand increase. The number of breathing cycles where the coefficient of variation of the respiratory rate was less than 4% is reported. Also, normalized averaged respiratory rate at cycle #0 and #100 are reported between brackets*.

Bio-inspired controller behavior as a function of metabolic demand is shown in Figures 8B,C. All but one condition was stable between 10 and 15 cycles after a metabolic perturbation. In the particular case of 80%-MBC17, the I_inc_ neuron behavior is slower than other SNNs. At cycle #100, bio-inspired controllers are synchronized and lead to a difference in respiratory rate of 3–8% when compared to non-injured models in the same metabolic demand condition. All results are stable at least for 300 s after controller activation and 180 s after metabolic perturbation.

## Discussion

This work demonstrates the potential benefit of using bio-inspired SNN controllers to restore impaired functions, e.g., impaired breathing ability with incomplete spinal cord injury. In addition, this work shows exploitation of hardware-in-the-loop (HL) real-time computation to facilitate efficient design and development of a closed-loop system in a multidisciplinary research environment.

The FPGA development of a closed-loop system benefits from the bio-inspired architecture. This has been demonstrated by the fact that an all-to-all connectivity pattern is not mandatory to achieve suitable performance. This bio-inspired controller is functional in open and closed-loop configurations with a connection density of 30.3 and 30.7%, respectively. The summary presented in Table 5 indicates that connection and neuron numbers vary widely across architectures. The biologically-based half-center or pattern generator architectures (Jung et al., 2001; Vogelstein et al., 2008; Molkov et al., 2014) have larger all-to-all density than those with feedforward architecture (Arena et al., 2010; Ayers et al., 2010), which are inspired by artificial neural network paradigms. We observe similar connection densities (29.9 ± 7.6%) to the biologically-based architectures when using 8-to-all connectivity. With our architecture, up to 1660 neurons can be implemented with the same multiplier resources and computation time-step by increasing the Spike computing phase and decreasing the idle phase. If hardware implementations used densities comparable to the computational network, then increasing the maximum number of connection per neuron to MaxC would result in a very large network. The networks presented in Table 5 have different ratios between the maximum numbers of connection C per neuron N. Therefore, a better understanding of the biological neural networks upon which we base our controllers may be as important as our ability to increase both neurons and MaxC resources to significantly improve closed-loop system functionality. The bio-inspired SNN controller functions appropriately with limited resources, as demonstrated in both open and closed-loop paradigms with perturbations (Figures 6–8). Table 3 reports the device utilization where only 5 multipliers and 19 18 kb-RAM-blocks are required. As mentioned earlier, the implemented system can extend its number of neurons up to hundreds without requiring additional resources. Moreover, the architecture presented in Figure 3 is easily scalable.

**Table 5:** **Literature review of connection densities for various spiking neural networks used in neuroscience or applicable controller research**.

		C (^*^ w/inputs)	**N**	**MaxC**	**All-to-all density (%)**	**MaxC-to-all density (%)**	**8-to-all density (%)**	**C/N**
Open-loop	This work	17	7	3	34.6	80.9	30.3	2.4
	Richter, 1996	16	6	4	44.4	66.6	33.3	2.6
	Ambroise et al., 2013	12	8	2	18.7	75.0	18.7	1.5
Closed-loop	This work	24 (^*^27)	10	4	24.0	60.0	30.0	2.4
	Molkov et al., 2014	30 (^*^33)	13	6	17.7	38.4	28.8	2.3
	Jung et al., 2001	12 (^*^18)	6	3	33.3	66.6	25.0	2.0
	Vogelstein et al., 2008	14 (^*^16)	4	4	87.5	87.5	43.7	3.5
	Ayers et al., 2010	31 (^*^44)	27	2	4.2	57.4	14.3	1.1
	Arena et al., 2010	32 (^*^46)	20	10	8.0	16.0	20.0	1.6
Average (std)		20.8 ± 8.3	11.2 ± 7.6	4.2 ± 2.4	30.2 ± 25.02	60.9 ± 22.0	27.1 ± 8.8	2.1 ± 0.7

*MaxC is the maximum number of connections C per neuron N in the network. All MaxC and 8-to-all densities were computed using the number of connections in the network vs., respectively, all-to-all neurons, MaxC-to-all and 8-to-all connections. Connections from external signals in closed-loop network were not included in the computation but are reported in brackets*.

This bio-inspired ventilatory controller simultaneously adapts and coordinates the breathing rate and pattern of the electrical drive over long timescales (breaths) with high computational power using short timescales (spikes). The intrinsic activity of the bio-inspired SNN controller is similar to respiratory networks (compare Figure 5A to results reported in Richter, 1996). The primary inhibitory oscillator is described as a coupled population of early-I and post-inspiration (or PI or post-I) neurons; these neurons are equivalent to the I_dec_ and E_dec_ neurons of the bio-inspired controller.

Surprisingly, a 10-neuron bio-inspired SNN also exhibits secondary behavior that is similar to that of the biological system: the double peak activity of the EI neuron. Yet, using a population instead of a single neuron could potentially improve the activity of others, such as Idec neuron. Indeed, the short duration of I_dec_ activity affects the duration of the EI second peak activity (longer than experimental measures). The functionality of the spike-based bio-inspired approach has been illustrated with various conditions. Bio-inspired controller activities were computed over an equivalent relatively long time (from 3 to 5 min) while showing good stability (coefficient of variation of respiratory rate remains under 5%). In addition, SNNs are known to be robust to noise or artifacts (Navalesi and Costa, 2003; Terzi et al., 2012). The capacity for stable regulation and dynamic properties of the biological system (Warren et al., 2014) are reproduced by the bio-inspired SNN controller, as shown in Figures 7, 8. These results demonstrate smooth synchronization with native breathing activity and robust responses to perturbations (turning ON the bio-inspired controller or sudden metabolic demand increase, for 3 examples of incomplete injury). In contrast with existing ventilatory assist systems that use electrical stimulation, the controller does not impose a fixed breathing rate. Also, the contribution of the electrical stimulation was appropriate and sufficient enough to improve respiratory rate despite disruptions in 3 injury models, reproducing the original desired breathing pattern.

One important aspect of this work is the hardware-in-the-loop (HL) real-time computational model. The spectrum of rehabilitation applications is wide, especially considering all possible contexts, but few HL models have been presented: epilepsy and Parkinson's disease (Detorakis et al., 2015; Ehrens et al., 2015). Experimental and clinical studies will always ultimately be required to validate the safety and functionality of closed-loop rehabilitation systems, but computational studies can be utilized as intermediary steps. Regulatory agencies are increasingly recognizing the potential contributions of simulation studies throughout the device development and evaluation process. One of the highest priorities for 2016 of the Center for Devices and Radiological Health of the US Food and Drug Administration is to develop computational modeling technologies to support regulatory decision making. Computational model results could be used as evidence to reduce the size of some clinical trials.

One important asset of the HL model is that it enables efficient investigation of controller performance under a wide range of conditions (physiological parameters and controller parameters). For example, we identified one of the main sensitive nodes in the bio-inspired SNN to regulate and synchronize the electrical drive with the native drive (Figures 6, 7A) prior to any animal experiment. HL results highlight strengths and weakness in the network architecture. Another example is the identification of the importance of adapting I_inc_ absolute synaptic strength potentials for the control of the electrical drive pattern: the I_inc_ related plasticity can improve the electrical drive behavior with sufficient resolution in real-time. However, the proposed closed-loop architecture does not provide high enough accuracy in this respect (Figure 7). The HL results advocate turning the single I_inc_ neuron into a sub-network, or population. The HL tool allows such development without compromising the other closed-loop functions. A third example is the independent implementation of active expiratory drive. The presented bio-controller does not include expiratory neurons identified in the literature (Richter, 1996; Molkov et al., 2014) but it still provides significant ventilatory assistance. Finally, the HL results call for further examination and development of learning rules. Learning rules in this work assist the breath depth and rate. Six similar sets of absolute synaptic strength potentials *P(inj)* were set to optimize the efficiency of the bio-inspired controller for each couple of injury level/metabolic demand increase (Figure 8). This HL computational study shows the need for a third learning rule related to the customization between the patient condition (injury level/ metabolic demand) and absolute synaptic strength potentials using a single SNN controller architecture. This third learning rule could be seen as equivalent to meta-plasticity in neuroscience. Learning rules could be easily implemented on the FPGA due to its scalable and modular structure at no additional time cost: updating all absolute synaptic strength potentials will be done during the kinetic phase, described in Figure 3B. Implementation of that third rule is the next step of this study combined with upcoming *in vivo* rat experiments.

In this study, we developed a rat computational model along with the accurate hardware implementation model to evaluate the feasibility of a bio-inspired controller for ventilation assistance in spinal cord injury applications. Each component of this model could be improved to more closely match experimental results. For example, the activation could include results reported from investigations of mechanisms of nerve electrical stimulation. Also, the metabolism model has a limited sensitivity: respiratory rate CV should not be higher than 0 at any time to represent breathing variability (Figure 8). Increasing the sensor diversity and number of sensors could improve the performance and versatility of the closed-loop system. Further insight from studies investigating the primary and secondary roles of various chemoreceptors (Ballantyne and Scheid, 2001; Huckstepp and Dale, 2011) could drive further enhancements to the bio-inspired system. For eventual implementation in humans, sensors will be required to gather reliable measures in a manner that minimizes inconvenience and limitations on activities such as speech. To measure the variables used in the simulation studies presented here, a small tube placed in or near the mouth or nose could be used to sample expired CO_2_; chest expansion sensors could be used to monitor volume. Further developments of sensor technologies would greatly facilitate clinical translation of adaptive ventilatory control systems.

## Conclusion

This innovative controller is inspired by neuroscienctific investigations and computational models of the respiratory neural network. Its SNN architecture has been designed to be functional and resource- efficient when implemented on an FPGA device while using neuron and kinetic models with low demands on computational resources. The closed-loop architecture takes advantages of the SNN structure to adapt the ventilatory control system with potentially numerous and varied types of feedback signals as illustrated by our custom hardware-in-the-loop real-time platform simulations. This work helps to design cross-disciplinary research experiments that pave the way to the design of the next generation of ventilation control systems.

## Author contributions

AZ developed the bio-inspired controller architecture and the SNN model. AZ and YB developed and tested the FPGA hardware. AZ and BH developed the HL models. RS prepared experimental materials and provided processed experimental data. AZ and SR analyzed simulated results. All authors contributed to the closed-loop concept for ventilatory assistance. SR, RJ, and JA supervised all aspects of the work. All authors discussed the results and commented on the manuscript.

### Conflict of interest statement

The authors declare that the research was conducted in the absence of any commercial or financial relationships that could be construed as a potential conflict of interest. The reviewer JR and the handling Editor declared their shared affiliation, and the handling Editor states that the process nevertheless met the standards of a fair and objective review.
